# The Dispersion and Coagulation of Negatively Charged Ca_2_Nb_3_O_10_ Perovskite Nanosheets in Sodium Alginate Dispersion

**DOI:** 10.3390/nano12152591

**Published:** 2022-07-28

**Authors:** Si Fu, Binbin Zhang, Zhiying Miao, Zhenyang Li, Rong Tu, Song Zhang, Bao-Wen Li

**Affiliations:** 1 State Key Laboratory of Advanced Technology for Materials Synthesis and Processing, Wuhan University of Technology, Wuhan 430070, China; fusi@whut.edu.cn (S.F.); mzy15527291106@whut.edu.cn (Z.M.); lzzyyy@whut.edu.cn (Z.L.); turong@whut.edu.cn (R.T.); kobe@whut.edu.cn (S.Z.); 2 State Key Laboratory of Silicate Materials for Architectures, School of Materials Science and Engineering, Wuhan University of Technology, Wuhan 430070, China; bbzhang@whut.edu.cn

**Keywords:** perovskite nanosheets, dispersion, charged polymer, aggregation of nanosheets, dynamic light scattering

## Abstract

Chemically exfoliated nanosheets have been extensively employed as functional nanofillers for the fabrication of polymer nanocomposites due to their remarkable electrical, magnetic and optical properties. However, achieving a good dispersion of charged nanosheets in polymer matrix, which will determine the performance of polymer nanocomposites, remains a challenge. Herein, we investigated the dispersion and aggregation behavior of negatively charged Ca_2_Nb_3_O_10_ (CNO) perovskite nanosheets in negatively charged sodium alginate (SA) aqueous dispersion using dynamic light scattering (DLS). When CNO nanosheets meet with SA, aggregation and coagulation inevitably occurred owing to the absorption of SA on nanosheets. By controlling the electrostatic attraction between positively charged poly(ethylene imine) (PEI) and negatively charged SA, the charge density and hydrodynamic size of SA can be tuned to enable the good dispersion of CNO nanosheets in SA. This result may provide a new strategy to achieve the good dispersion of charged nanosheets in charged polymers for the rational design of multifunctional nanocomposites.

## 1. Introduction

Chemically exfoliated inorganic nanosheets have attracted extensive attention due to their prominent physical and chemical properties [1]. For practical applications, functional nanosheets are usually combined with polymers to fabricate high-performance nanocomposites [2]. However, the large surface energy of nanosheets and incompatibility between inorganic fillers and polymer leads to the aggregation of nanosheets in the polymer matrix, which all contribute to degradations in the performance of nanocomposites. Therefore, good dispersion of nanosheets is essential to achieve high-performance nanocomposites [3,4,5,6].

Since the electrostatic repulsion, a large number of charged nanosheets, such as graphene oxide [7,8], oxide nanosheets (e.g., Ca_2_Nb_3_O_10_ and Ti_0.87_O_2_) [9,10], 2D transition metal carbides and/or nitrides [11], and nanoclay [12,13] have exhibited dispersibility in solution. Furthermore, liquid crystals consisting of chemically exfoliated nanosheets such as niobates, titanates and clays received a lot of attention due to their unique electric, magnetic, and mechanical properties [14,15,16].Therefore, charged nanosheets with a high surface charge density and excellent physicochemical properties are favorable to prepare nanocomposites. In addition, the affinity between inorganic fillers and polymer matrix is another critical factor that determines the dispersion of nanosheets in polymers. Phase separation in nanocomposites will be suppressed to a large extent due to the good affinity of nanosheets, ensuring their homogeneous dispersion in the polymers. In recent years, the ionic interactions between charged nanoparticles and polymer were investigated by theory and simulations to improve the dispersibility of nanoparticles in polymers [17,18]. By taking advantage of the ionic interactions between titanoniobate nanosheets and polydopamine, their compatibility was improved and the good dispersion of nanosheets in nanocomposites was achieved by Hu and co-workers [19]. Fernandes et al. improved the affinity of between inorganic fillers and polymer through the ionic interactions, achieving the good dispersion of SiO_2_ nanoparticles in polymers [20]. Nevertheless, polymer chains will adsorb on the nanosheet surfaces owing to the interaction between them, resulting in the aggregation of nanosheets [21]. While previous work mostly focused on the coagulation mechanism of charged nanosheets, how to avoid their aggregation has rarely been reported.

In this work, we discussed the dispersion and coagulation of negatively charged CNO perovskite nanosheets in SA using DLS. The charge density and hydrodynamic size of SA were controlled by adjusting the weight ratio of SA to PEI. When the weight ratio of SA to PEI was 0.045, the Zeta potential of SA increased from −62 mV to −53.8 mV, and the average hydrodynamic size of SA decreased from 730.9 to 199.8 nm. By controlling the charge density and hydrodynamic size of sodium alginate, the average hydrodynamic size of CNO nanosheets in SA dispersion decreased from 5.2 μm to 352 nm, achieving a good dispersion of charged nanosheets in polymer dispersion.

## 2. Materials and Methods

### 2.1. Main Materials

CNO perovskite nanosheets were synthesized by liquid-phase exfoliation according to a previous report [22]. SA was purchased from Sigma Aldrich. Branched poly(ethylene imine) (PEI, average M_w_ = 70,000) was obtained from Aladdin Company (Shanghai, China). Ultra-pure water collected from a Milli-Q system was used in all experiments.

### 2.2. Dispersion of CNO Nanosheets in SA/PEI Dispersion

SA powder was dissolved in ultra-pure water at a concentration of 2 wt% under stirring for 12 h. PEI solution (1 wt%) was dropwise added to sodium alginate dispersion (1 wt%) in different weight ratio of PEI to SA with stirring, including 0, 0.045, 0.1, 0.25, 0.5, 0.75, 1. After stirring 5 h, SA/PEI dispersion with different weight ratios of PEI to SA were prepared. The CNO nanosheet dispersion was diluted to a concentration of 2 wt% in ultra-pure water. Then, the CNO nanosheet dispersion was dropwise added to SA/PEI dispersion to obtain CNO/SA/PEI dispersion with 0.03 wt% CNO nanosheet.

### 2.3. Characterization

AFM images of CNO nanosheets were recorded using atomic force microscope (Asylum research Cypher ES, Santa Barbara, CA, USA). The Zeta potential and hydrodynamic size of SA/PEI and CNO/SA/PEI dispersion were measured using Zetasizer (Nano ZS-90, Malvern Instruments Ltd., Westborough, MA, USA).

## 3. Results and Discussion

### 3.1. Characterization of CNO Perovskite Nanosheets

**Figure 1:**
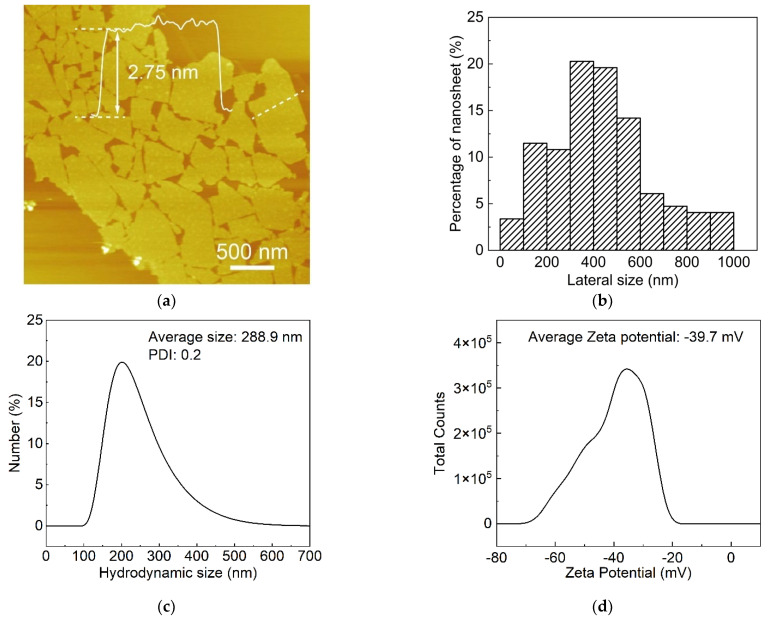
Characterization of CNO nanosheets. (**a**) AFM image of CNO nanosheets and height profile of an individual nanosheet across the white line; (**b**) size distribution of CNO nanosheets (about 1000 samples were counted); (**c**) Zeta potential distribution; (**d**) hydrodynamic size distribution of Ca_2_Nb_3_O_10_ nanosheets in water.

CNO perovskite nanosheets were obtained by the liquid-phase exfoliation of layered compound KCa_2_Nb_3_O_10_. As shown in Figure 1a,b, the atomic force microscopy (AFM) observation of CNO nanosheets revealed the uni-laminar feature of the CNO nanosheets, with a thickness of about 2.75 nm and an average lateral size of approximately 0.5 µm. For further evaluating the size distributions of the CNO nanosheets, DLS was utilized to evaluate the hydrodynamic radius of CNO nanosheets in ultra-pure water. The average hydrodynamic radius of CNO nanosheets in the analyzed aqueous solution was about 0.3 µm, which was significantly lower than the lateral size observed by AFM. This can be attributed to the fact that the scattering behavior of nanosheets is different to that of spherical particles, which is commonly used for DLS analysis [23]. Although DLS cannot provide quantitative information, it can still reflect the size of nanosheets [24]. Figure 1c indicated that the average Zeta potential of CNO nanosheets in ultra-pure water was about −39.7 mV, confirming that CNO nanosheets have a high negative charge density. The strong electrostatic repulsion between individual CNO nanosheets enabled the homogeneous dispersion of CNO nanosheets in water.

### 3.2. Formation of SA/PEI Dispersion

**Figure 2:**
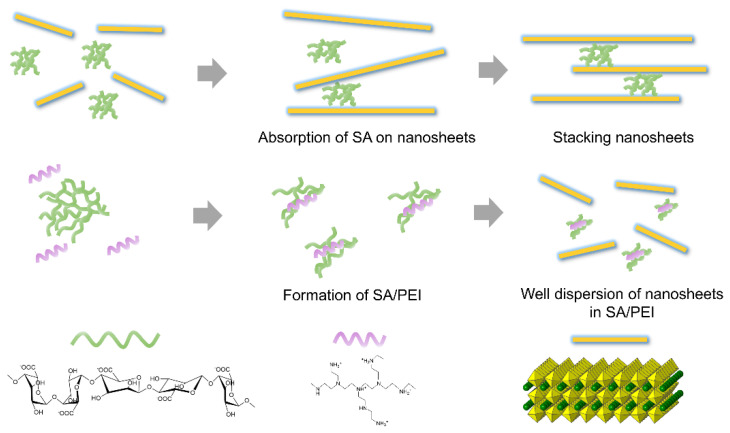
Schematic illustration of the adsorption of negatively charged SA on negatively charged CNO nanosheets and the formation of SA/PEI.

As shown in Figure 2, one typical negatively charged polyelectrolyte, SA, exhibited high negative charge density because of abundant carboxy groups (COO^−^) on its molecular chains. In contrast, PEI is a typical positively charged polyelectrolyte owing to the high number of protonated ammonium groups decorated with its molecular chains. When PEI was added to SA dispersion, the PEI molecular chain tended to adsorb on SA due to the electrostatic attraction between anionic SA and cationic PEI [25]. The interaction energies of hydrogen bonding and pi-pi stacking are distributed between 25–40 and 1–50 kJ/mol, respectively, while that of ionic interactions is more than 100 kJ/mol [26,27]. The carboxyl groups of SA and the amino group of PEI are complementary ionic structures, resulting in a synergistic effect of hydrogen bonds and ionic interaction [28]. In terms of Coulomb’s law, electrostatic interaction energies depend on the dielectric constant of the charged particles and the distance between charged particles. However, the dielectric constant of charged polymers is related to the type of polymer and temperature. The dielectric constant values of polyethyleneimine (PEI) and sodium alginate (SA) are approximately 9 and 10, respectively [29,30], which are much larger than other polymers. Thus, there is a strong electrostatic attraction between PEI and SA.

**Figure 3:**
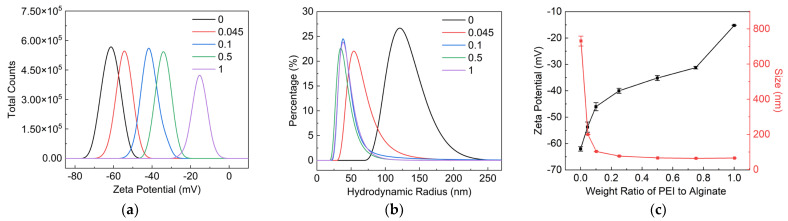
Characterization of SA/PEI dispersion with weight ratio of PEI to SA. (**a**) Zeta potential, (**b**) hydrodynamic size distribution and (**c**) average Zeta potential and hydrodynamic size of SA/PEI dispersion.

Figure 3a,c indicated that the Zeta potential of PEI/SA increased with the increase in the weight ratio of PEI to SA. The increase in the Zeta potential of PEI/SA demonstrated the decline in negatively charged carboxy groups on SA molecular chain, implying the adsorption of PEI on SA. Figure 3b,c showed the hydrodynamic size distribution and average hydrodynamic size of PEI/SA with a different weight ratio of PEI to SA. The average hydrodynamic size of SA dispersion was about 683 nm. The molecular chain of SA tended to twin around each other, owing to the hydrogen bonding between a large number of hydroxyl and carboxyl groups, contributing to the relatively high hydrodynamic size of SA in water. The hydrodynamic size of PEI/SA decreased with the increase in the weight ratio of PEI to SA. This result suggests that the electrostatic attraction and hydrogen bonding between PEI and SA will weaken the interaction between the molecular chains of SA. SAs with a smaller hydrodynamic size was further obtained with the aid of shear force during the mechanical stirring process.

### 3.3. Dispersion of CNO in SA/PEI

**Figure 4:**
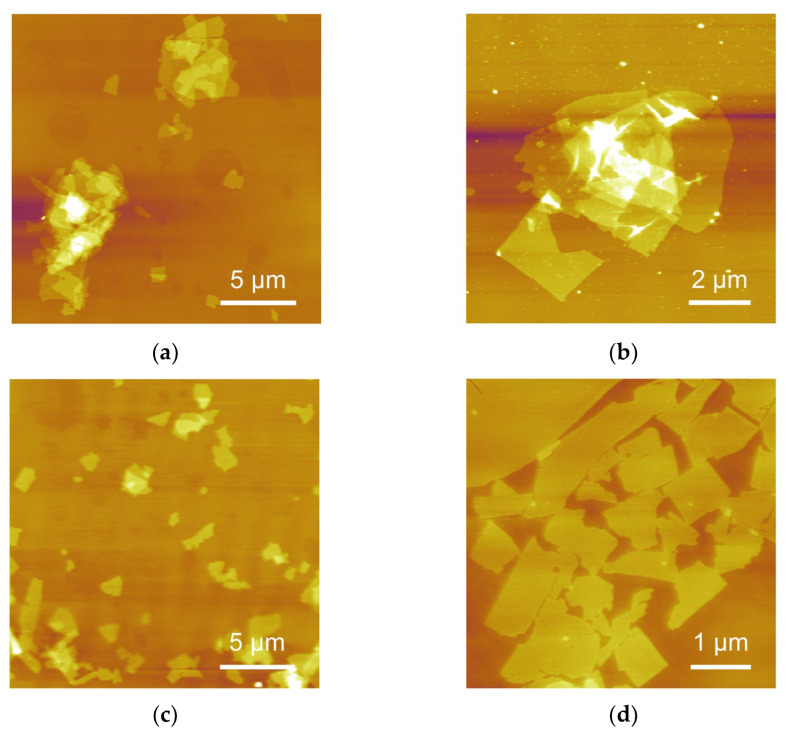
AFM characterization of CNO nanosheets transferred from (**a**,**b**) CNO/SA and (**c**,**d**) CNO/SA/PEI dispersion (the weight ratio of PEI to SA is 0.045).

AFM images of CNO nanosheets transferred from CNO/SA and CNO/SA/PEI dispersions are depicted in Figure 4. As shown in Figure 4a,b, negatively charged SA led to the stacking of CNO nanosheets. One part of the SA molecular chain adsorbed on the nanosheets surfaces, with the other part of SA molecular chain extending around to adhere to neighboring nanosheets. In this way, the SA molecular chain served as a bridge connecting adjacent nanosheets, leading to the coagulation of CNO nanosheets. Therefore, it is essential to prevent the absorption of SA molecular chain on CNO nanosheets to suppress their aggregation.

The adsorption behavior of polymer moleculars on the surface of CNO nanosheets largely depended on the charge type, charge density, molecular weight and size of polymer [31]. With the same molecular size, cationic polymers exhibited a higher absorption ability for negatively charged capacity than anionic polyelectrolytes due to the electrostatic attraction. Electrostatic repulsion between anionic polymer and negatively charged nanosheets suppressed the adsorption of the polymer chain on CNO nanosheets [32]. Moreover, the charge density and particle size of polymers in dispersion had a significant impact on the absorption behavior, which was directly related to the aggregation of nanosheets. The polymer with a higher molecular weight and larger particle size at certain dispersions contained more functional groups, which acted as absorption sites for CNO nanosheets. Hence, reducing molecular weight and size in dispersions was an effective way to prevent the absorption of SA on the CNO nanosheets. Driven by the electrostatic attraction between PEI and SA, the PEI molecular chain adsorbed on the SA molecular chain; therefore, the hydrodynamic size of SA decreased, significantly reducing the number of absorption sites of SA molecular chain. As shown in Figure 4c,d, CNO nanosheets could be homogeneously dispersed in the SA/PEI dispersion. The stacking of CNO nanosheets rarely appeared in CNO/SA/PEI dispersion.

**Figure 5:**
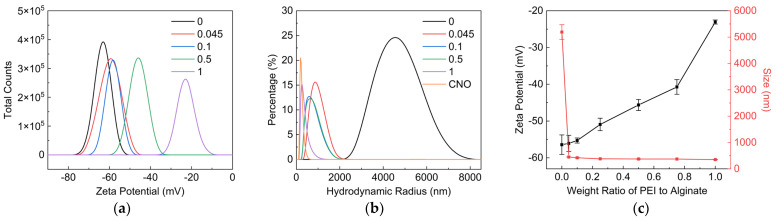
Characterization of CNO/SA/PEI dispersion with weight ratio of PEI to SA. (**a**) Zeta potential, (**b**) hydrodynamic size distribution and (**c**) average Zeta potential and hydrodynamic size of CNO/SA/PEI dispersion.

The Zeta potential of CNO nanosheets with different weight ratios of PEI to SA was demonstrated in Figure 5a,c. The Zeta potential of CNO/SA/PEI dispersion increased with the increasing weight ratio of PEI to SA. With the addition of CNO nanosheets, the Zeta potential of SA dispersion increased from −62.1 to −56.4 mV as the ratio of PEI to SA was zero, above which the Zeta potential of CNO/SA/PEI dispersion reduced compared to SA/PEI dispersion. The absolute value of Zeta potential reflects the stability of dispersion. The dispersion with larger absolute value of Zeta potential exhibited better stability. Hence, we can conclude that the stability of SA/PEI was enhanced by adding CNO nanosheets. The size distribution of CNO nanosheets in SA and SA/PEI dispersion was measured using DLS. As shown in Figure 5b, the size of the CNO nanosheets in SA/PEI dispersion decreased with the increase in the ratio of PEI to SA, finally approaching to the dispersion state in the ultra-pure water at the PEI/SA ratio of 1. As shown in Figure 5c, the average hydrodynamic size of CNO nanosheets in SA dispersion was approximately 5.2 μm, further confirming their coagulation. In contrast, there was no obvious increase in hydrodynamic size of CNO nanosheets in PEI/SA dispersion, implying the suppressed aggregation of CNO nanosheets in PEI/SA dispersion.

## 4. Conclusions

In conclusion, a novel strategy was developed to improve the dispersion of charged nanosheets in charged polymer. By means of electrostatic attraction between negatively charged SA and positively charged PEI, the Zeta potential and hydrodynamic size of SA were adjusted. Furthermore, with the interaction between SA and PEI, the adsorption of SA molecular chain on CNO nanosheets can be suppressed, contributing to the dispersion of CNO nanosheets in SA dispersion.

## Data Availability

The data supporting the findings of this study are available by request to the corresponding author.
